# Prenatal maternal stress and offspring aggressive behavior: Intergenerational and transgenerational inheritance

**DOI:** 10.3389/fnbeh.2022.977416

**Published:** 2022-09-23

**Authors:** Ngala Elvis Mbiydzenyuy, Sian Megan Joanna Hemmings, Lihle Qulu

**Affiliations:** ^1^Department of Basic Science, School of Medicine, Copperbelt University, Ndola, Zambia; ^2^Division of Medical Physiology, Biomedical Science Research Institute, Stellenbosch University, Cape Town, South Africa; ^3^Department of Psychiatry, Faculty of Medicine and Health Sciences, Stellenbosch University, Cape Town, South Africa

**Keywords:** aggression, stress, epigenetics, sexual aggression, neuromodulation, prenatal stress, intergenerational inheritance, transgenerational inheritance

## Abstract

Even though studies have shown that prenatal maternal stress is associated with increased reactivity of the HPA axis, the association between prenatal maternal stress and fetal glucocorticoid exposure is complex and most likely dependent on unidentified and poorly understood variables including nature and timing of prenatal insults. The precise mechanisms in which prenatal maternal stress influence neuroendocrine signaling between the maternal-placental-fetal interface are still unclear. The aim of this review article is to bring comprehensive basic concepts about prenatal maternal stress and mechanisms of transmission of maternal stress to the fetus. This review covers recent studies showing associations between maternal stress and alterations in offspring aggressive behavior, as well as the possible pathways for the “transmission” of maternal stress to the fetus: (1) maternal-fetal HPA axis dysregulation; (2) intrauterine environment disruption due to variations in uterine artery flow; (3) epigenetic modifications of genes implicated in aggressive behavior. Here, we present evidence for the phenomenon of intergenerational and transgenerational transmission, to better understands the mechanism(s) of transmission from parent to offspring. We discuss studies showing associations between maternal stress and alterations in offspring taking note of neuroendocrine, brain architecture and epigenetic changes that may suggest risk for aggressive behavior. We highlight animal and human studies that focus on intergenerational transmission following exposure to stress from a biological mechanistic point of view, and maternal stress-induced epigenetic modifications that have potential to impact on aggressive behavior in later generations.

## Introduction

Prenatal maternal stress can arise from malnutrition, major life events, bereavement, catastrophic events, depression, or anxiety. Hans Selye described stress as a physiological response pattern that occur in response to a stressor whether external or internal and will last if the stimulus persists (Selye, 1956). The stress response is a homeostatic process, and implicated in this response is the neuroendocrine system called the hypothalamic-pituitary-adrenal (HPA) axis, which regulates several physiological processes including energy expenditure and storage, digestion, the immune system, mood, and emotional responsivity to stress (Herman et al., 2016) through the release of glucocorticoids. Response to acute stress does not result in long-term changes of the stress response axis, whereas chronic stress can exert effects that may be quite long lasting (Godoy et al., 2018b). A dysregulation of the HPA axis caused by repeated or extreme exposure to stress is associated with elevated cortisol levels and may be linked to anxiety and depression pathology (Menke, 2019). Prenatal maternal stress increases fetal plasma cortisol to levels that overwhelm the metabolic capacity of placental 11β-hydroxysteroid dehydrogenase, the enzyme that normally protects the fetus from the higher levels of maternal glucocorticoids by converting cortisol to the inactive cortisone (Słabuszewska-Jóżwiak et al., 2020). Increased circulating fetal cortisol levels bind to both glucocorticoid and mineralocorticoid receptors that are expressed in high levels in multiple fetal brain regions, including the limbic system, hypothalamus, and cortex, where it impacts on neurogenesis, gliogenesis and synaptogenesis, suggesting its role in influencing cognitive, behavioral, and morphological development (De Kloet and Meijer, 2019). These structures have been implicated in aggressive behavior, a phenomenon defined by Moyer (1971) as an “overt behavior that has the intention of inflicting physical damage on another individual.”

There has been a growing body of research supporting the suggestions that offspring are affected by prenatal parental trauma exposures, and possibly even prior to their conception (Yehuda and Lehrner, 2018). While it is now accepted that severe stress exposure in a parent is a risk factor for several behavioral disorders, in offspring, the perspective originated debatably in the 1960s following the observation of behavioral and clinical problems in offspring of Holocaust survivors (Felsen, 1998). This phenomenon now termed the intergenerational effect of trauma suggests that exposure to adverse events have deleterious impact on individuals with the potential of their offspring manifesting effects of the traumatic exposures of their parents (Yehuda and Lehrner, 2018). In other words, it suggests that the effect of the experience of trauma is “passed” somehow from one generation to the next. Intergenerational transmission suggests transmission of the effect of these experience from the parent (F0) to the first/second filial generation (F1/F2) while transgenerational transmission describes transmission from third filial generation (F4) to the fourth filial generation (F4) (Crews et al., 2007). For the last quarter of a century, the mechanisms by which these long-term effects are sustained have been a concern among scientists. While evidence on the involvement of the hypothalamic-pituitary-adrenal and disruption of the intrauterine environment due to changes in uterine arterial blood flow have been described, advances in neurosciences and molecular biology have facilitated our understanding of epigenetic mechanisms i.e., changes in gene expression without changes in DNA sequences-that occur in parents because of exposure to chronic stress and are transmitted to the offspring (Breton et al., 2021). Results from studies suggests that beside the established pathways for psychiatric disorders: environment, genetics, and gene-environment interactions, there exist mechanisms by which maternal stress influences expression/modification of genes involved in stress/behavior regulations that is transmitted to offspring (Kundakovic and Jaric, 2017; Boyce et al., 2020). However, it remains unclear how such changes persist and are transmitted across generations.

This review examines studies suggesting associations between prenatal maternal stress and risks of offspring aggressive behavior in offspring, as well as the possible mechanisms of transmission of prenatal maternal stress response effects to the fetus: (1) maternal-fetal HPA axis dysregulation; (2) changes in uterine artery blood flow; (3) epigenetic modifications of genes implicated in aggressive behavior. We also discuss the concepts of intergenerational and transgenerational transmission and narrow our review on epigenetic inheritance to better understands the mechanism(s) of transmission from parent to offspring. We begin this review by discussing studies showing associations between maternal stress and alterations in offspring taking note of neuroendocrine, brain architecture and epigenetic changes that may suggest risk for aggressive behavior. We endeavor to highlight animal and human studies that focus on intergenerational transmission following exposure to stress from a biological mechanistic point of view, and maternal stress-induced epigenetic modifications that impact on generations later.

## Prenatal maternal stress and fetal brain development

The prenatal period is a developmental period in which the fetus is most vulnerable to alterations in both external and internal environments, with effects lasting over longer periods of time. In short, it is a period in which the fetus is programmed (Almond and Currie, 2011). During this programming period, the development of the brain which relies on cutoff cellular, molecular and transcriptional events is most vulnerable to wiring for later-life behavioral disorders (Miller et al., 2014; Spiers et al., 2015). Lee et al. (2019) in a large-scale study showed that the expression of genes and their variants linked to the development of several brain disorders peak during pregnancy. An “optimal” intrauterine environment is necessary for “healthy” fetal brain development. Disruptors of this environment which include among others nutrition, illness, and stress, will affect fetal neurodevelopment.

The stress experienced by a pregnant mother, referred to as prenatal stress may include poor dieting, illness, or could be ongoing events of life, like violent neighborhood, ongoing wars (chronic stress), or sudden changes in a woman’s daily routine or environment including earthquakes (Coussons-Read, 2013). Maternal prenatal stress occurs in coincidence with the period of rapid brain cell formation, development, differentiation, and establishment of circuits and synapses. Disruption of these vital processes as result of exposure to prenatal stress was shown to increase the development of emotional and behavioral problems in children in later life (van den Heuvel et al., 2021). For instance in a longitudinal case-control study (Wu et al., 2020a) observed that the presence of congestive heart failure (CHD) was higher in 65% of pregnant women who experienced stress during pregnancy compared to only 27% of pregnant women who did not experience significant stress during pregnancy. Brain imaging studies have shown that prenatal maternal stress is associated with significant thinning of the cortex and disruption of functional and structural connections between frontal cortex, the hippocampus and the amygdala (De Asis-Cruz et al., 2020; Wu et al., 2020b). Despite these studies being conducted on smaller sample sizes and many confounders could not be controlled, the observed brain changes in offspring following exposure of mothers to stress prenatally, do suggests an association with prenatal stress. After all, epidemiological studies have shown evidence generational effects of stress on birth weight and cardiovascular disorders in humans (Veenendaal et al., 2013; Pembrey et al., 2014). The transmission of these stress response effects could occur through several mechanisms.

## Mechanisms of transmission

Several mechanisms have been suggested to be responsible for the observed outcomes of prenatal stress on offspring. One is the transfer of genes that are vulnerable to stress from mother to offspring (Bowers and Yehuda, 2016) and the other is the direct impact of the traumatic stress on the mother affecting her ability to parent her child and the altered childhood environment (Yehuda et al., 2001). Studies using recent molecular techniques have claimed that the effects of prenatal stress are passed to the offspring through non-genomic, mechanisms affecting DNA function or gene transcription (Ferguson-Smith, 2011; Ferguson-Smith and Bourc’his, 2018).

## Changes in uterine artery blood flow

One mechanism by which prenatal maternal stress might affect the fetus is through the changes in the uterine arterial blood flow to the fetus (Ridder et al., 2019). Though prenatal maternal stress has been shown to evoke cardiorespiratory changes in both mother and fetus, in baseline and laboratory-induced stress in several studies, these cardiorespiratory changes seem to exist almost independent of one another (Walsh et al., 2019). Few studies have described associations between maternal stress indicators and fetal heart rate (DiPietro et al., 2008a). Using a Doppler ultrasound, uterine blood flow can be measured. Measures of high uterine artery resistance indicated by presence of a notch in the ultrasound waveform pattern have been linked with underweight for full term babies (Bower et al., 1993; Harrington et al., 1996). While one study showed no associative relationship between maternal stress and uterine blood flow in women in the 20th week of gestation (Kent et al., 2002), another study indicated a significant positive association between maternal stress levels and both maximum artery resistance scores and mean artery resistance (Teixeira et al., 1999). DiPietro et al. (2008b) investigated the transmission of maternal psychological states to the fetus, by monitoring maternal-fetal pairs during the 32nd week of gestation for maternal ECG, and respiration as well as fetal heart rate and movement in a laboratory-induced relaxation study. They observed significant reduction in all maternal physiological measures including salivary cortisol levels apart from respiration baseline to relaxation. Fetal heart rate and movement also declined significantly from baseline to relaxation, while fetal heart variability increased. Despite variability in the results, the authors suggested that the fetal responses to some extent reflect fetal perception of changes in the intrauterine environment (Kinsella and Monk, 2009). *In utero*, prenatal stress conditions prime the uterine environment to prepare the fetus for postnatal life. Transmission of maternal stress–based changes may mold fetal behavior because of *in utero* perception and learning and may have relevance for fetal brain development, and ultimately, offspring outcomes. Despite studies implicating the HPA axis in increasing resistance to uterine artery blood flow, norepinephrine however has been suggested as the likely mediator *via* activation of the sympathoadrenal system (Teixeira et al., 1999). Starkman et al. (1990) measured peripheral noradrenaline levels in patients with and without pheochromocytoma and observed an associative relationship between plasma noradrenaline concentration and anxiety. This has been corroborated by animal studies implicating noradrenaline in the reduction of blood flow velocity and pressure in the uterine artery of pregnant guinea-pig (Fried and Samuelson, 1991; Hu et al., 2021). There is however scanty research in the maternal-fetal stress pathway on uterine blood flow and later life offspring psychopathology.

## Maternal-fetal hypothalamic-pituitary-adrenal axis dysregulation

The fetal HPA axis is fully developed and functional by the twenty-second week of pregnancy. Even at this stage it is still vulnerable and sensitive to environmental stimuli and assaults (Challis et al., 2001; Duthie and Reynolds, 2013; Sheng et al., 2021). Both animal and human studies have confirmed that the stress response is mediated by the hypothalamic–pituitary–adrenal (HPA) axis, which results in release of corticotrophin-releasing factor and the eventual secretion of glucocorticoids (Meaney, 2001). Alterations in the HPA axis and changes in CRF neuro-functions induced by prenatal stress have been shown to be relatively persistent (Heim et al., 2000). Even though the main role of this HPA axis is to mediate the appropriate endocrine response to stress, prolonged exposure to elevated levels of glucocorticoids has damaging effects on the developing brain, which may secondarily lead to behavioral problems later in life (Sapolsky et al., 1990; Champagne et al., 2008). Most of the observed behavioral alterations in mothers with pregnancies conceived during periods of traumatic stress and even experiences of chronic stress prior to conception, have been shown to be transmitted to the offspring and to the subsequent generation (Brunton, 2013). Even though the mechanism(s) by which the effects of maternal stress is/are passed from the mother to fetus is unclear, fetal HPA axis response, maternal glucocorticoids as well as maternal behavior have been implicated (Del Giudice, 2012).

Increased circulating maternal glucocorticoids because of prenatal stress, exposes the fetus to excessive glucocorticoids. This has been proposed as one way by which maternal stress effects are passed to the offspring (Thomas et al., 2018). A support to this suggestion is from a study in which pregnant rats that were treated with a synthetic glucocorticoid produced offspring with characteristics similar to rats that were exposed to prenatal stress (Welberg et al., 2001). In addition, removal of maternal adrenal gland relieves some of the stress effects in the offspring (Barbazanges et al., 1996). Despite these findings, the involvement of maternal glucocorticoids can be argued on two basis: first, 11β-hydroxysteroid dehydrogenase type 2 (11b-HSD2) an enzyme that metabolizes corticosteroids into inactive form, cortisone and therefore limits the fetal exposure to active maternal glucocorticoids is highly expressed in the placenta (O’Donnell et al., 2012; Reynolds, 2013). Second, despite increased productions of maternal glucocorticoids in response to stress during late pregnancy, the levels are not higher compared to virgin female rats; the highest levels of corticosterone reached do not exceed the peak diurnal levels observed in gestation (Price et al., 2019).

Studies of prenatal stress in animals have shown lower levels of expression of 11β-HSD2 mRNA and decreased 11B-HSD2 activity, both of which are associated with increased 11β-HSD2 methylation in the placenta (Nugent and Bale, 2015). These changes would have significant repercussion on amount of maternal glucocorticoid the fetus is eventually exposed to, leading to activation of fetal HPA axis. Despite this, it is still uncertain the role maternal glucocorticoids play in the effect maternal stress has on the offspring. However, the timing of elevation of the circulating glucocorticoid levels may be critical, as the peaking of its level during the nadir of the circadian rhythm, may contribute to *in utero* programming of the fetus (Cottrell and Seckl, 2009). Important to note is the fact that, by embryonic day 16, adrenal glands of the fetus are well-developed and secrete glucocorticoids, suggesting that maternal stress may activate fetal HPA axis to secrete glucocorticoids, that could be responsible for fetal brain programming (Cottrell and Seckl, 2009). Beside glucocorticoids playing a key role in maternal stress transmission from mother to the offspring, catecholamines have also been suggested to be involved, thus adrenalectomy would not only remove glucocorticoid but also removes catecholamines, mineralocorticoids, and sex steroids, which may also be involved in programming of the fetus. Compared to non-pregnant rats, stress-induced adrenaline release is reduced in late pregnancy in pregnant rats. Despite this reduction, the responses due to noradrenaline are maintained as a result of sympathetic nervous system activation (Parker and Douglas, 2010). Just as with glucocorticoids in which the placenta expresses enzymes that metabolize them, the placenta also express monoamine oxidase and catechol-*O*-methyltransferase that breakdown maternal noradrenaline. However, up to 12% of maternal noradrenaline can be transferred to the fetus, which may have detrimental effects on the fetus (Nandakumaran et al., 1983). Increased circulating levels of maternal catecholamines cause placental vasoconstriction, leading to decreased blood through the placenta and therefore impairing oxygen and nutrient delivery to the fetus. These restrictions provoke the fetal HPA axis and activate fetal sympathetic-adrenomedullary stress responses, which may contribute to the HPA axis’ role in fetal programming and expression of aggressive-like behavior in the adult offspring (Otten et al., 2001).

Historically, using animal and human studies, the HPA axis markers had been used to explain the potential for prenatal maternal stress to program fetal brain development through placental alterations, but more recently epigenetic measures have opened up new avenues for better understanding (Babenko et al., 2015; Deodati et al., 2020; Murray et al., 2021). Epigenetic mechanisms have been implicated in stabilizing genomic expression across the lifespan in response to a variety of environmental cues (Provençal and Binder, 2015). They have so far been regarded as key mediating mechanisms responsible for long-lasting stress-related dysregulation that affect the brain and modify behaviors.

## Concept of intergenerational and transgenerational transmission

It has been generally accepted for decades that DNA is the only heritable component that defines a phenotype; however, there has also been evidences of involvement of epigenetic components in determining phenotypic inheritance (Tricker, 2015). Epigenetic modifications are changes to the DNA without change to the DNA building block sequence. They regulate the turning off and on of genes (Hornschuh et al., 2021). The generational transmission of these modifications defined as “observable epigenetic changes that are transmitted from one generation to the next, or from parent’s generation, named the zero filial generation (F0), to the offspring’s generation, named the first filial generation (F1)” is considered intergenerational transmission (Heard and Martienssen, 2014), while transmission from the F2 generation of F3 without exposure to the initial environmental stressor or F3 to F4 with intrauterine exposure to stress (Stenz et al., 2018). The plethora of existing definitions of epigenetics makes the definition of transgenerational epigenetics difficult. Researchers have classed epigenetics into context-dependent and heritable modifications (Crews, 2008). Context-dependent modifications are mitotic in nature, occur in body cells and “persist only for the duration of the lifetime of an organism” (i.e., within generation) (Crews, 2008). Heritable epigenetic modifications are meiotic in nature and occur in the germline (across generation). To be considered a germ-line dependent epigenetic inheritance, (Crews, 2008) and Skinner (2008) strongly suggests that the exposure of one generation to an adverse event which is encountered by the subsequent generations should be the first criterion to be considered for generational inheritance. Then the number of subsequent generations after the initial exposure will follow to delineate intergeneration from transgeneration. Based on this, intergenerational changes seen in the F1 and F2 generations are not necessarily heritable, since some of the modifications found in the subsequent generations may be attributed to the direct effects of germline exposure. It is therefore worth stating that an exposed female rodent or human (F0), pregnant with fetus (F1), the germline of the fetus is also exposed to the adverse event (F2). Intergenerational inheritance hence is described as transmission from F0 to F1/F2 and F1 to F2, while transgenerational inheritance in females is described from F3 generation onward in females and from F2 in males (Heard and Martienssen, 2014). On this basis, generational epigenetic inheritance is the passing of markers of epigenetics from one generation to another resulting in observed phenotypic changes (Kovalchuk, 2012; Perez and Lehner, 2019).


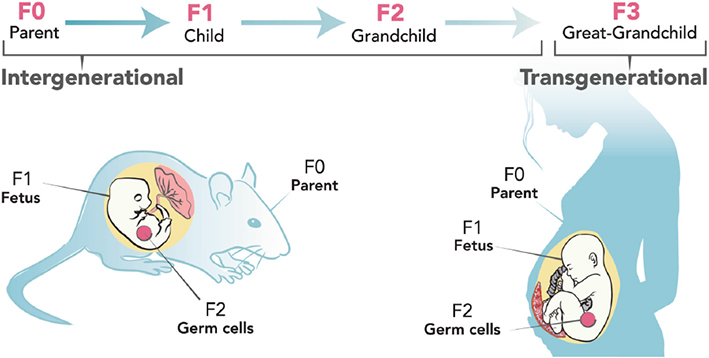


An exposed female rodent or human (F0), pregnant with fetus (F1), the germline of the fetus (F2). Intergenerational inheritance depicted from F0 to F1/F2 and F1 to F2, while transgenerational inheritance in females is described from F3 generation Adapted from Breton et al. (2021).

The concept of generational transmission of the effects of stress from parents to offspring started finding itself in medical literature in the 60s following observation of behavioral and clinical psychopathologies in offspring of Holocaust survivors (Scharf, 2007). Individuals who experienced the Holocaust lived with psychopathologic disorders and post-traumatic stress disorder years and even decades afterward. These effects were even observed in the second and third generation of survivors. Some authors described this phenomenon the “second generation” pain describing the experience of these children. Their description focused more on the feeling of guilt and absorbed trauma from the effects that their parents live with. In the early 60s there was a knowledge void, to describe the chronic effects of traumatic stress and the observations of these effects on offspring. Children of families impacted by the Holocaust, shared feelings of psychological overburden of the Holocaust experience as though they were present at the time (Sigal and Rakoff, 1971). While at that time, there was absolutely no clue on mechanisms by which these effects were observed in the children of Holocaust survivors, it is important to applaud the establishment of the diagnosis of PTSD in the late 70s and early 80s as a principal driver of investigations in the transmission of stress effects across generations. PTSD diagnosis asserted that the exposure to adverse traumatic events would lead to physiological and behavioral changes that could persist long after the adverse event (Gangi et al., 2009). Weinfeld et al. (1981) recounted that it was Rakoff’s, earlier works, which sparked off academic and clinical research into the transmission of the psychological effects of the Holocaust experiences from survivors to their offspring Comprehensive and up-to-date bibliography can be found in the works of Sigal (1998), Kellermann (2008), Gangi et al. (2009), Barel et al. (2010), Shrira et al. (2011), and Dashorst et al. (2019).

Rakoff’s publication generated much empirical research with several articles getting published with varied efforts attempting to corroborate clinical manifestations of psychopathologic disorders observed in offspring to the traumatic stress experienced by parents during the Holocaust. The psychopathologic disturbances in offspring included dysphoria, anxiety, traumatic nightmares, and depression. Any attempt to link the observation on the offspring to the Holocaust treatment were popularly refuted citing methodological flaws but leaning toward the potential damaging effects of such claims on certain subgroups (Van IJzendoorn et al., 2003; Sagi-Schwartz et al., 2008). It was noted that several of these studies did not consider the psychopathology of the parents but assumed that the offspring manifestation were solely because of parental exposure. As several literature were churned out in attempt to describe this, distinction was gradually made between the offspring’s reaction to symptoms in parents versus the manifestation in the offspring as a direct result of a psychopathologic disorder in the parent (Schwartz et al., 1994). Despite several literature suggesting family dynamics, social psychology and attachment theory as possible explanations for the observation of behavioral abnormalities in offspring of Holocaust survivors (Kellerman, 2001), other studies documented a higher prevalence of mood and anxiety disorders and PTSD in offspring of Holocaust survivors compared to children of parents who did not directly experience the traumas of the Holocaust (Hogman, 1998; Shrira et al., 2011). Subsequent studies directly assessed *via* clinical interviews, parents who experienced the Holocaust established a relationship between PTSD in offspring and their own parents (Yehuda et al., 1998). Yehuda et al. (2008) reported that later studies also confirmed the increased prevalence of PTSD in offspring of Holocaust survivors in response to their own traumatic exposures was associated with maternal PTSD in Holocaust survivors.

Several occurrences around the globe since the Holocaust have brought to bear the phenomenon of psychopathologic manifestations in offspring of traumatic stress experienced by their parent (s). These include PTSD and social behavioral disorders found in offspring of Australian Vietnam Veterans, the 9/11 attacks, the Armenian Genocide of 1915, apartheid in South Africa, wars, and ethnic cleansing such as the genocide in Rwanda (Astourian, 1990; Westerink and Giarratano, 1999; Dobson, 2009; Kim et al., 2021). A study to investigate the mental health of children whose mothers were exposed to the Rwandan genocide refuted the theory of trans-generational trauma transmission (Roth et al., 2014). The 1994 genocide in Rwanda claimed the lives of 10% of the Rwanda population (Schaal and Elbert, 2006). Several decades after, the prevalence of PTSD has stayed high in Rwanda (Pham et al., 2004). To correlate the psychopathological status of the offspring generation to the parental psychopathology resulting from past traumatic experiences, Hagengimana et al. (2003) interviewed 125 Rwandan survivors of the 1994 genocide and their 12-year-old children to assess maternal PTSD symptoms and aggressive and antisocial behavior in the children. PTSD in these mothers was not associated with aggressive and antisocial behavior in the children, a finding which negates or stupefies the assumption of generational transmission of effects of traumatic stress. The authors agree that violence in domestic settings increases risk of mental health disorders but suggested that it was childhood exposure to domestic violence by the mothers not maternal PTSD that was associated with their children’s aggressive and antisocial behavior.

Some researchers have noted that the association of observed behavioral or biologic changes in offspring to the adverse events in the parents may simply mean both generations have similar genetic risks, rather than an intergenerational transmission of the behavioral or biological effect. This perspective aligns more with conventional genetic model of trait inheritance but falls short of explaining the inheritance of the observed effects of stress. While the term “intergenerational transmission” is often used loosely in literature to imply the totality of parental biological processes that directly impact on the offspring, it is quite complexly challenging to differentiate whether it is parental biology or behavior that mediates these effects in the offspring. It is however difficult to distinguish between parental biology and parental behavior as mediators of offspring effects. Bates et al. (2018) explained this by suggesting that parents do serve as behavior models in which children emulate and tend to respond to changes within their milieu in manners reminiscent of their parents, hence the behavioral effect may not necessarily have molecular intergenerational undertone. Vostanis et al. (2006), had earlier posited that behavioral changes observed in offspring can occur because of child rearing deficits on the part of the parent. Bowers and Yehuda (2016) also noted that it is possible for offspring to report the adverse traumatic experiences as narrated by their parents or imagine the traumatic experience of their parents, adding that offspring behavior may then be influenced by these, and not necessarily transmission from parents to offspring.

The conviction that behavioral changes observed in offspring as result of biological changes in the parent exposed to adverse traumatic events received wings from results from a large cohort study that examined the effects of starvation stress in pregnant women during the Dutch famines (Barker, 1990). Discussions from these studies provided unique scenarios of what can be described as “generational transmission,” where offspring were directly affected by deprivation of nutrients in the mother, which to larger extents were from stress-related alterations in maternal behaviors. Decades of studies correlating infant morbidity and later adulthood mortality in England and Wales (Barker and Osmond, 1986), Norway (Waaler, 1984), and Finland (Kaplan and Salonen, 1990) suggested that rather than focusing on genetic transmission of traits which did not address the observation of certain behavioral and psychiatric features in offspring not found in parents, attention should be redirected toward the intrauterine environment and factors that may alter its normal physiology. The Dutch study which investigated prenatal exposure to the Dutch famine, when food intake was reduced to 500–1,500 kcal per day during the period of October 1944–May 1945, observed reduced newborn weight, head circumference and birth length as well as maternal weight (Stein and Susser, 1975). Studies on effect of exposure of pregnant women to traumatic stress during Vietnamese war (Pless Kaiser et al., 2012) and the 1992–1995 War in Bosnia and Herzegovina (Fatušiæ et al., 2005) on infant health parameters suggested that nutrient deficiency impact on fetal physiology changing their structure and metabolism, and hence alter brain development. These changes may be the sources of later life psychiatric and behavior-related disorders. While the debate on methodology and conceptual interpretation raged on, it is worth noting that stress effects that are inherited *via* an “generational transmission” mode evoke morphological, neuroendocrine, and epigenetic changes in the offspring.

## Changes in the offspring

A growing body of research demonstrates that inhibitory circuitry in the brain is directly and persistently affected by prenatal stress. Several lines of evidence from animal models as well as human studies, suggest that prenatal stress interferes with fetal development, ultimately determining changes in brain maturation and function that may lead to the onset of neuropsychiatric disorders (Marchisella et al., 2021; Roshan-Milani et al., 2021). While some studies may not directly link prenatal stress and aggressive behavior in later life there are however studies that show that early childhood stress is linked to aggressive behavior. Other studies show alteration of structures and circuits that have been implicated in aggressive behavior.

## Neuroendocrine changes

Several animal and human studies have determined levels of stress markers in blood, saliva, and urine of stressed pregnant mothers to assess HPA-axis activity. While it is established that prenatal maternal stress impacts on pregnancy with potential on affecting the developing fetus it is important to note that impact of this depends on the gestational period (Sandman et al., 2012). Exposure to stress during the first trimester increases risk of miscarriage, while exposure during later periods of gestation increases risk for low weight at birth (Cabrera et al., 1999; Abramova et al., 2021). This programming that occurs during these periods, particularly impacts neuroendocrine (HPA-axis) responses. The dysregulation of the HPA axis because of exposure to stress during pregnancy may also interfere with other systems, including, the hypothalamic–pituitary–gonadal axis as well as the vasopressinergic and oxytocinergic systems. This is evidenced by alterations in steroidogenesis, and impaired social behaviors in prenatally stressed offspring. This programming also impacts on maternal behavior with potential of negative phenotypes to be transmitted to future generations (MacLeod et al., 2021).

Brunton and Russell (2010) reported increased adrenocorticotrophic hormone and corticosterone secretion levels in socially defeated pregnant rats exposed to resident lactating rats during the 5 days of the last week of pregnancy. Male offspring displayed increased aggressive-like behavior in a resident-intruder test while female offspring rats did not show marked changes compared to control rats. Similar studies have reported greater expression of glucocorticoid receptor mRNA and corticotropin-releasing hormone mRNA in the central amygdala in male and female rat offspring of prenatally stressed mothers compared to controls (Price and McCool, 2022). These studies suggest that prenatal stress programs the HPA axis and aggressive-like behavior in male and female rat offspring differently. Diminished glucocorticoid feedback mechanisms in the limbic system may underlie the HPA axis hyper-reactivity to stress in offspring of prenatally stressed female rats (Brunton et al., 2015). Increased expression of CRF mRNA in the bed nucleus of the stria terminalis (BNST) and central amygdala, but not the PVN in rats (Funk et al., 2006; Jiang S. et al., 2019) and activation of CRF R2-positive neurons in the medial amygdala (Fekete et al., 2009; Ivanova et al., 2021) may explain the manifestation of aggressive behavior in the offspring, as Haller (2018) explained that rivalry aggression and hyperarousal-driven aggression are controlled by the medial amygdala while predatory aggression is controlled by the central amygdala and hypoarousal-associated violent aggression recruits both nuclei. It is important to note that central CRF administration into the lateral ventricle or lateral septum reduces maternal aggression in rodents (Rosinger et al., 2020; De Guzman et al., 2021), suggesting that decreased CRF neurotransmission during lactation is thought to contribute to the attenuated stress response in dams as well as promoting maternal aggression (Gammie et al., 2004; Bosch et al., 2018). Murgatroyd and Nephew (2013) showed that prenatal chronic social stress does not only disrupt maternal behavior but also impair maternal care of offspring. This was corroborated by reduced expression of oxytocin receptor (OXTR), prolactin receptor (PLRL), and vasopressin (AVPR1a) in brain areas implicated in social bonding. They have furthered demonstrated that these effects are not only limited to the F1 generation as they observed increased corticosterone and neuroendocrine alterations in the F2 generation (Carini and Nephew, 2013; Nephew et al., 2017). Social stress also reduced levels of these hormones in the brain and periphery explaining the inefficient lactation observed in the adult female F1 generation (Nephew and Bridges, 2011).

To determine if the effects of prenatal stress in the F1 generation will be transmitted to the F2 generation without intervention through maternal line, Brunton and Russell (2010), Grundwald and Brunton (2015) mated prenatally stressed female rats and F1 control rats with control males and housed them under non-stress conditions throughout pregnancy. They recorded marked increase in levels of corticosterone and ACTH in F2 females of prenatally stressed mother compared to the controls. Like the studies of Jiang Y. et al. (2019) these observations were associated with increased expressions of CRF mRNA expression in the paraventricular nucleus and reduced hippocampal glucocorticoid receptor mRNA expression. Attenuation of HPA axis responses and hippocampal glucocorticoid mRNA expression was less in male F2 offspring of prenatally stressed females. These F2 males of prenatally stressed females exhibited increased aggressive-like behavior in a resident-intruder test compared with F2 control males. Increased expression of CRF and CRF receptor mRNA in central amygdala of F2 male offspring of prenatally stressed female rats compare to the controls was suggested as reasons for the heightened aggressive-like behavior. The F2 females showed no significant difference with the controls. De Souza et al. (2013) investigated the long-lasting effects of repeated prenatal restraint stress in the last 7 days of pregnancy on aggression and social behavior of the offspring. Male offspring of prenatally stressed mothers reared by prenatally stressed mothers showed increased anxiety-like and aggressive-like behavior. These observations were associated with oxytocin and arginine vasopressin neurons in the paraventricular nuclei. On the other hand, male offspring of prenatally stressed mothers reared by prenatally non-stressed mothers showed reduced arginine vasopressin neurons compared to the male offspring of prenatally non-stressed mothers reared by prenatally non-stressed mother. Prenatal stress may increase greater environmental sensitivity which may be both an opportunity and risk factor for aggressive behavior.

Human studies have also provided evidence of offspring neuroendocrine changes following prenatal maternal stress. De Bruijn et al. (2009) compared the patterns of cortisol in a group of preschool children that were exposed prenatally to maternal stress to a non-exposed group and found that girls of prenatally exposed mothers showed higher cortisol levels compared to non-exposed girls but not in boys, corroborating animal studies which suggest that prenatal maternal stress may be impact HPA-axis activity differently for males and females. In a prospective longitudinal cohort study of mothers and children in which measures of anxiety and depression were recorded during pregnancy and the postpartum period to examine the long-term association between prenatal maternal stress and diurnal cortisol at age 10 years, researchers observed significant association between prenatal maternal stress and infant cortisol levels at age 10, accounting for aggressive behavioral risk also observed in this cohort of children (O’Connor et al., 2005). These studies suggest that prenatal stress is associated with long-term disturbance in hypothalamic-pituitary-adrenal (HPA) axis function. Fetal programming hypotheses which opine that state that early development of the HPA-axis of the children may have been affected (O’Connor et al., 2005; Egliston et al., 2007; Howland et al., 2017; Rajyaguru et al., 2021). These underline the associations between prenatal maternal stress and offspring behavior including aggression. In a study to investigate Holocaust-related maternal age of exposure and PTSD and association with offspring ambient cortisol and PTSD-associated symptom expression, ninety-five Holocaust offspring and Jewish comparison subjects, received diagnostic and psychological evaluations, and assayed for 24 h urinary cortisol by radioimmunoassay (RIA). The study showed that levels of cortisol and PTSD-associated symptom expression in the offspring of Holocaust survivor were related to the age at which Holocaust victim was exposed, with the greatest effects associated with increased age at exposure (Lehrner et al., 2014). The association of low cortisol levels and other neurobiological alterations in offspring with the risk factor of maternal PTSD, has also been corroborated by the findings of Yehuda et al. (2007), Castro-Vale et al. (2020), and Yehuda et al. (2009). *In utero* glucocorticoid programming due to exposure to the traumatic events was suggested as reason for the observed alterations. Cross-talking between the hypothalamic–pituitary–adrenal (HPA) and hypothalamic–pituitary–gonadal (HPG) axes have also been suggested as basis for prenatal maternal stress associated childhood aggression. Nguyen et al. (2018) studied the relationship between baseline testosterone (a marker for HPG axis function) and cortisol (a marker for HPA axis response) and their association with aggressive behavior in 11-year-olds whose mothers during pregnancy were exposed to the 1998 Quebec ice storm. They found that only at lower levels of subjective prenatal maternal stress, baseline testosterone and cortisol reactivity were positively correlated. Cortisol reactivity mediated the link between testosterone and aggression. The results of their study gave strength to the views that prenatal maternal stress influences fetal HPA–HPG interactions, which may be associated with aggressive behavior in late childhood.

Prenatal maternal stress has also been shown to greatly impact fetal development of the monoaminergic system. This system includes norepinephrine (NE) dopamine and serotonin (5HT). The brain levels of these neurotransmitters are regulated by the enzyme monoamine oxidase (MAO), of which there are two isoforms MAO-A and MAO-B (Kumar et al., 2021). These enzymes are expressed during fetal development and play a role in fetal brain development, with MAOA expressed in significant levels more than MAOB (Wang et al., 2011). Fetal MAOA promoter interactions with adverse environmental alterations like prenatal stress have been linked to later-life behavioral disorders (Mentis et al., 2021). There is overwhelming evidence that the manifestations of aggression are linked to MAOA activity, an observation confirmed by Cases et al. (1995) and Godar et al. (2016) who demonstrated that MAOA KO mice showed increased aggressive behavior in comparison to wild-type littermates. Frau et al. (2022) also showed that inhibition of MAO during intrauterine development resulted in manifestation of aggressive behavior in offspring.

Brain structural studies in adult rodents have linked projections from the cortex to the amygdala to play important roles in emotional behavior, suggestion interruptions or disruptions of this pathway is linked to aggressive behavior (Mcdonald, 1998; McHale et al., 2022). Brain imaging studies conducted in humans by Barrash et al. (2022) and Grafman et al. (1996) and a systematic review of neural, cognitive, and clinical studies of anger and aggression by Richard et al. (2022) have also provided more evidence that disruption of the frontocortico-amygdalar circuit as well as ventromedial frontal lobe lesions are linked to aggressive behaviors. The monoaminergic system particularly the 5-HT system has been greatly implicated in the formation of this circuit and other circuits subserving emotional behavior (Rainnie, 1999; Desrochers et al., 2022). At the molecular level prenatal maternal stress has been shown to influence amygdala expression of 5-HT1A receptors as well as GABAergic function (Ehrlich et al., 2015). Marchisella et al. (2021) investigated the effects of prenatal maternal stress on the expression of genes related to glutamatergic and GABAergic neurotransmissions in male rat offspring prefrontal cortex and the amygdala, two areas implicated in aggression and other emotional behaviors. They observed that prenatal maternal stress fostered an imbalance in the excitatory and inhibitory pathways in the prefrontal cortex by altering the expression of glutamatergic-related genes. In the amygdala, prenatal maternal stress shifted the excitation-inhibition balance toward inhibition by affecting the transcription of GABAergic-related genes. Their findings suggest that the excitation-inhibition imbalance of the prefrontal cortex-to-amygdala transmission may be a long-term signature of prenatal maternal stress and may increase the risk for aggressive behavior upon more stress. GABA-ergic neurotransmission within the mPFC has been suggested to be critical in the emotions and aggression-related activities of the amygdala (Suzuki and Tanaka, 2021). mPFC sends exciting inputs to the amygdala and is a target of incoming outputs from the amygdalae. More studies have showed a correlation between the strength of the endogenous connections between the right amygdala and mPFC, and have further suggested that mPFC GABA content predicts variability in the effective connectivity within the mPFC-amygdala circuit, providing new insights on emotional physiology (Delli Pizzi et al., 2017). Involvement of these neuropeptides as mediators of prenatal stress and adult offspring aggressive behavior were also investigated by Ehrlich et al. (2015) who studied the effects of prenatal stress and antidepressant treatment on gene expression related to GABAergic and serotonergic neurotransmission in the amygdala. Other monoaminergic neurotransmitters implicated in aggressive behavior and influenced by prenatal maternal stress during fetal brain development are dopamine and norepinephrine. Prenatal stress has been shown to disrupt the development, differentiation, and integration of frontal cortex dopaminergic neurons. (Long-Term Effects of Prenatal Stress on Dopamine and Glutamate Receptors in Adult Rat Brain) showed that prenatal stress increased the expression of dopamine (D2) receptors in the medial prefrontal cortex, dorsal frontal cortex, hippocampal CA1 region as well as the nucleus accumbens, implicating prenatal stress in the disruption of the corticolimbic pathway, which plays a major role in emotional and social behavioral disorders including impulsive aggression (Mapping the neurocircuitry of impulsive aggression through the pharmacologic review of anti-impulsive aggressive agents). Extensive coverage of the role of the monoaminergic system in aggression can be found in reviews by Godar et al. (2016), Korzan et al. (2001), and Mentis et al. (2021).

## Brain morphological (neuroanatomical) changes

The endocrine and physiological responses to stress that result in long-term behavioral changes, have been shown to be connected to significant brain structural and neuronal alterations. As noted above, stress activates the HPA axis, which in turn excites nuclei of the locus coeruleus in the brainstem to release norepinephrine which does not only prepare the system for alertness and attention, but it also initiates the encoding of information linked to the stress event (Ross and Van Bockstaele, 2021). Stress excites the limbic system and indirectly through the locus coeruleus which communicates with the amygdala and directly through exciting corticotrophin releasing factor and glucocorticoid receptors expressed in the hippocampus, amygdala, and prefrontal cortex, altering neuron shape, the process of formation of new neurons, neuron dendrite, and spine morphology (Smith and Pollak, 2020). Connecting structural cellular changes following chronic stress to behavioral alterations has been the efforts of decades of research using animal models and advancing technology in human studies.

More attention has been dedicated to understanding the impact of prenatal maternal stress on brain morphological development for the past decades. In a study to examine the influence of prenatal maternal stress on the size and shape of the corpus callosum Coe et al. (2002), disturbed pregnant monkeys using acoustic disturbance for 10 min per day from GND 90 to GND 140. MRI scans of infants’ brain sagittal and coronal views showed decreased overall size of the corpus callosum in male offspring of prenatally stressed mothers compared to infants born of undisturbed pregnant mothers. Results of their study however showed increased size of the corpus callosum in female offspring of prenatally stressed mothers, once again testifying of the differential effects of prenatal stress on males and females. Their findings agree with the observations of Tanaka-Arakawa et al. (2015) perinatal factors influence the development of the corpus callosum in infancy and early adulthood, possibly affecting communication between the hemispheres. The corpus callosum serves as a structural connection between the left and right brain hemispheres relaying signals that are critical in the emotion and aggressive behavior (Schutter and Harmon-Jones, 2013). This connectivity between both hemispheres Functional interhemispheric connectivity has been proposed as a possible mechanism that could explain the frontal cortical asymmetry of anger and aggression. Disruption of the back-and-forth information relay between both hemispheres *via* the corpus callosum together with the concept of frontal cortical asymmetry, have been suggested to contribute to anger and aggression (Hofman and Schutter, 2009).

In a prospective longitudinal study Buss et al. (2010), assessed the impact of prenatal maternal stress at 19-, 25-, and 31-weeks gestation on infant brain morphology. MRI scans of the offspring when they were between ages six to nine years, the researchers observed reductions in gray matter density after correcting for other confounding variables including postpartum perceived stress. Their findings showed that stress at week 19 of gestation was significantly associated with reduction in gray matter density in the premotor cortex, lateral temporal cortex, the prefrontal cortex, and the medial temporal lobe. Gray matter volume reduction in these areas have been associated with increased aggressive behavior (Hofhansel et al., 2020). The studies of Schoretsanitis et al. (2019) and Wong et al. (2020) in which they assessed gray matter variations in eighty-four patients with schizophrenia spectrum disorders, for aggressive behavior. After the required corrections for confounding variables, their results showed that verbal aggression was associated with gray matter volume in left inferior frontal gyrus. Similar significant gray matter volume reductions in the amygdala, insula, and orbitofrontal area, were observed in fMRI scans of individuals with intermittent explosive disorder relative to healthy individuals. This study particularly correlated hyperactivity in the left insular with composite aggression scores in these individuals with intermittent explosive disorder (Seok and Cheong, 2020).

Larger right amygdala volume in offspring girls but not boys was associated with increased levels of maternal cortisol during their early but not late pregnancy in a study to investigate the correlation between stress during different periods of pregnancy and hippocampi and amygdala volumes in offspring at 7 years of age. There was however no correlation between offspring hippocampi volume and prenatal maternal cortisol levels in either sex (Buss et al., 2012). In another study, to determine if altered brain connectivity was the connection between prenatal maternal stress and child aggressive behavior, Hay et al. (2020) examined MRI scans 54 human mother-child dyads using fractional anisotropy and mean diffusivity. Stress during the third trimester was positively associated with weaker connectivity of the amygdala-frontal pathway, which mediated third trimester stress symptoms and manifestations of aggressive behaviors in the male child (Hay et al., 2020). Findings from animal and human lesion studies have implicated the amygdala and orbitofrontal cortex in aggressive behavior (McCloskey et al., 2016; Ibrahim et al., 2021).

## Epigenetic modifications of genes implicated in aggressive behavior

For the last quarter of a century, the mechanisms by which these long-term effects are sustained have been a concern among scientists. While evidence on the involvement of the hypothalamic-pituitary-adrenal and changes in the uterine artery blood flow have been described, advances in neurosciences and molecular biology have facilitated our understanding of epigenetic mechanisms that occur in parents because of exposure to chronic stress and are transmitted to the offspring. Acute as well as chronic stress has been shown to evoke changes in gene expression by producing specific signals to DNA, chromatin and mRNA that do not alter the nucleotide sequence (Keverne et al., 2015). Deoxyribonucleic acid (DNA) contains heritable information coded in nitrogenous bases, that is transmitted from parents to offspring through the process of transcription. Following transcription, the transcript is modified, and the non-coding portions called introns are sliced out and the coding portions called exons are converted to messenger RNA (mRNA) which are used as a template for protein synthesis in the process called translation (Shi and Manley, 2015). This process of gene expression is regulated at different levels; during the process of DNA copying, transport of the transcript to the cytoplasm, during and after protein synthesis, as well as epigenetically (Guerra et al., 2015) and by a wide range of external factors like environment (oxygen levels, temperature, humidity, daylight cycles, and nutrients etc.), internal signals such as stress hypoxia, nutrients, inflammation and even genetic non-coding RNAs (Dhama et al., 2019). Epigenetic modifications to the DNA include DNA methylation, genomic imprinting, histone modifications, and non-coding RNAs (Wei et al., 2017). These processes influence the accessibility of the gene to transcription factors by affecting the packaging of DNA and the structure of chromatin, thus “switching on or off” the genes (Larochelle et al., 2018). There is evidence that stress in general induces epigenetic changes that affect neuronal development, synaptic connections, neuronal function, and ultimately influencing behavioral adaptation to stress (Godoy et al., 2018a; Jiang Y. et al., 2019). These may be the connection between environmental cues and dysregulation of the HPA axis following stress.

One study has shown that increased DNA methylation of the HSD11B2 promoter in the placenta associates with altered newborn behavior, with poorer infant quality of movement, a marker of adverse neurobehavioral outcomes (Marsit et al., 2012). This methylation will reduce placental buffering function against enhanced levels of stress hormones during maternal stress. Observation from animal studies have shown that epigenetic modification of specific exon 17 in the promoter region of the glucocorticoid receptor (GR) gene (NR3C1) is linked to prenatal maternal stress and GR expression in brain regions including the hippocampus (Weaver et al., 2004; Szyf et al., 2005; Gans and Coffman, 2021; Knox et al., 2021). One study in rats, showed that the NR3C1 promoter region is regulated through the quality of prenatal maternal stress and maternal care of the offspring (Szyf et al., 2005). This epigenetic programming of GR expression suppresses HPA activity, which may in part explain how prenatal maternal stress influences HPA axis stress response in the offspring (Van Bodegom et al., 2017). Radtke et al. (2011) observed increased methylation of NR3C1 promoter in infants whose mother’s experienced traumatic stress during their pregnancy. Oberlander et al. (2008) also found that maternal exposure to stress during the third trimester was positively correlated with increased methylation of NR3C1 in newborns and increased salivary cortisol stress responses at 3 months. In a similar study Hompes et al. (2013) examined 83 pregnant women, and assessed daily cortisol levels to evaluate maternal stress during the course of pregnancy to establish a relationship between prenatal maternal stress during pregnancy and methylation levels of the NR3C1 promoter region. CpG9 sites methylation was significantly associated with prenatal maternal stress. Methylation at the 1F exon in the offspring was influenced by prenatal maternal stress only during the first two trimesters. However, Van Der Knaap et al. (2014) reported a non-significant effect of perinatal stress, on NR3C1 methylation in adolescent, in a study in which they examined the impact of various types of stress including prenatal stress on methylation of NR3C1 in the blood of a population sample of 468 adolescents. The variations in findings may be explained based on methodologies used or individual differences in the potential to of offspring to develop behavioral disorders. There is therefore the need for longitudinal, comparative studies to examine the variations in the NR3C1 methylation status, at specific CpG sites alongNR3C1 and NR3C1 regulatory elements, and how these variations in methylation associate with offspring outcomes. Higher levels of methylation at the NR3C1 promoter region were observed in teenage boys and girls of mothers who encountered incidences of violence from their partners during pregnancy but not before are after the pregnancy (Radtke et al., 2011). In another study, involving 20 years-old adolescents of pregnant mothers who were exposed to the Rwanda genocide while pregnant with them, also showed higher levels of methylation the NR3C1 promoter with corresponding lower levels of serum cortisol, in comparison to same aged children of mothers who were not exposed to the genocide. The children of the exposed mothers were also noted to display symptoms of PTSD and tendencies of aggressive behaviors. This goes to further suggest that epigenetic changes in abnormal activities of the HPA axis are transmitted across generations (Perroud et al., 2014). These observations are consistent with those made in newborns of mothers in the Democratic Republic of Congo who experienced severe traumatic stress, during the war in the then Zaire (Veling et al., 2013). The findings of Kertes et al. (2016), adds evidence to the observed widespread effects of chronic stress and war trauma on methylation of key genes regulating the HPA axis (CRH, CRHBP, NR3C1, and FKBP5). Significant methylation at transcription factor binding (TFB) sites, were suggested to be unique to chronic or war stress.

The FKBP5 gene, which encodes the FK506 binding protein 5 (FKBP5), a heat shock protein 90 undergoes epigenetic modification following stress (Fries et al., 2017). FKBP5 interacts with NR3C1 as a co-chaperone of the glucocorticoid receptor complex, regulating the sensitivity of the glucocorticoid receptor by modulating intracellular glucocorticoid signaling and plays an important role in homeostatic regulation of the stress response (Hartmann et al., 2021). Interactions of FKBP5 gene variants and life stressors increase the risk for mood and aggressive disorders, as well as other diseases phenotypes (Binder, 2009; Engelhardt et al., 2021). Stress has been shown to increase levels of *FKBP5* expression by changing the DNA methylation patterns at the *FKBP5* locus. In mice exogenous steroid administration modified DNA methylation in *FKBP5* within brain (Lee et al., 2010). A study involving Holocaust survivors reported elevated levels of cytosine methylation within the *FKBP5* gene in survivors of the Holocaust and their adult offspring in comparison to control offspring (Yehuda et al., 2016). Their results showed that holocaust exposure influenced FKBP5 methylation at bin 3/site six in the Holocaust survivors as well in their offspring. They observed that, methylation at this site was higher in Holocaust survivors compared to control subjects. This FKBP5 methylation was associated with wake-up cortisol levels. This study is among the first study to demonstrate an association between prenatal maternal traumatic stress with epigenetic changes in both exposed parent and offspring, demonstrating the intergenerational effects of psychological trauma. Variants of the FKBP5 genes have been linked with aggressive behavior mediated by hypothalamic-pituitary-adrenal axis dysregulation. In a cross-sectional study involving 583 Italian prisoners at the Penitentiary District of Abruzzo-Molise in central Italy Bevilacqua et al. (2012), sort to determine the interaction between four different variants in FKBP5 (rs3800373, rs9296158, rs1360780, and rs9470080) and childhood trauma in predicting aggressive behavior observed a significant influence increased expression of the *FKBP5* on aggressive and violent behavior in jail in individuals exposed to childhood trauma. This suggests that interactions between variants of *FKBP5 gene* with early life stress increase the risk of aggressive behavior.

Also implicated in moderating the link between prenatal maternal stress and aggressive behavior disorders is the serotonin transporter (SERT) gene polymorphic region (5-HTTLPR) coded by the SLC6A4 gene (Oberlander et al., 2010; McKenna et al., 2021). This transporter clears the synaptic cleft of serotonin by re-uptake into the presynaptic neuron. Persistent changes in SLC6A4 gene expression patterns, have been shown to correlate with mood and aggressive behavior disorders. This may also be another mediator between prenatal maternal stress and aggressive behavior. Wankerl et al. (2014) examined 83 CpG sites in the CpG island of the SERT gene for the effects of prenatal and traumatic stress on *in vivo* expression of SERT mRNA in 133 healthy young adults. They observed that the reduction in expression of SERT mRNA were unrelated, to the prenatal stress. However, Sjaarda et al. (2017) reported that stressed pregnant Slc6a4 + / + and Slc6a4 ± mice and pup brains showed high levels of methylation profiles several genes affecting neuron development and cellular mechanisms implicated in aggressive behavior. This further supports the suggestions that prenatal stress increase the risks of developing mood and aggressive disorders in offspring of mothers with deficits in serotonin related pathways. In a related human study DeLano et al. (2020), determined levels of DNA methylation of NR3C1, SCG5, and SLC6A4 genes in 53 pregnant mothers experiencing various forms of prenatal stress including community-level deprivation and individual stresses. They reported that higher levels of methylation at 8 CpG sites in SLC6A4 but not methylation of NR3C1 was positively related to maternal community-level deprivation. The differential methylation of key genes in the serotonin pathway have also been associated with childhood aggression (Jiang S. et al., 2019). Measuring brain serotonin using positron emission tomography (PET) Wang et al. (2012) reported lower serotonin synthesis in the orbitofrontal cortex of adult males with high childhood-limited aggression. These observations were supported by increased methylation of SLC6A4 promoter in peripheral white blood cells of individuals with physical aggression during childhood. Hemmings et al. (2018) found the STin2 VNTR12 allele to be associated with high levels of appetitive aggression but low levels of reactive aggression in a study that investigated the role of genetic variants in the serotonin transporter (SLC6A4; 5-HTTLPR, rs25531, and STin2 variants) genes in the etiology of appetitive aggression in 290 South African Xhosa males. This is not the case in the study of Muench et al. (2019) in which they found no association between methylation of the SLC6A4 promoter and threat-related amygdala activation in individuals with alcohol dependence or controls.

## Discussion and conclusion

The aim of this review article was to bring comprehensive basic concepts about prenatal maternal stress and mechanisms of transmission of its to the fetus. In this review we considered prenatal stress to relate to such conditions that occur preconception and during pregnancy. We also briefly discussed the concept of intergenerational and transgenerational transmission of stress effects, with focus on epigenetics. We narrowed our review on epigenetic modifications of genes that have been described to influence aggression by highlighting studies both in animals and humans. We reviewed literature on impact of prenatal stress on offspring brain morphology, neuroendocrine systems, and epigenetic modifications, as we attempt to connect these to currently described brain structures and pathways that sub-serve various forms of aggression in animals and humans. Fetal brain development is a sensitive period in which neuron proliferation, differentiation, migration, and aggregation, occur for the entire gestational period are mainly genetically determined and epigenetically directed (Cattane et al., 2020). Even though studies have shown that prenatal maternal stress is associated with hyperactivity of the HPA axis, the relationship between prenatal maternal stress and exposure of the fetus to fetal glucocorticoids is complex, non-linear, and most probably controlled by yet unidentified and poorly understood variables including nature and timing of prenatal insults.

We have provided literature and research showing support for transgenerational epigenetic inheritance, and its possible role in later life aggressive behavior, it however is not clear the specific effects of transgenerational epigenetics on offspring behavior given the undescribed role of confounding factors. Even as we implicate epigenetic changes in offspring behavior from adverse maternal exposures during pregnancy, it is important to factor in the possible role(s) of posttranslational modifications to non-histone proteins on brain development which in the strict sense of it may not reflect true epigenetic reprogramming events (Gabory et al., 2011). It is thus imperative to conduct longitudinal studies over multiple generations with recurrence of frequent measures of aggressive behavior including information for both parents for us to begin to comprehend the mechanisms that underlie intergenerational transmission of stress effects that increase risk of aggressive behavior in later life. This will guide and inform us in distinguishing the roles played by genetic and non-genetic mechanisms in transmission of prenatal maternal stress effects and thus help us understand interactions between genetic and non-genetic factors. In such studies delineating impact of parenting and offspring aggressive behavior from consequences of transgenerational inheritance, and examining if aggressive behavior in the F3 on F2 prenatal exposures (F1) or childhood experiences will further broaden our understanding of the mechanisms of transmission.

Use of animal models to identify how changes in epigenetic modifications in the germline affect development of the offspring, increasing the risk for aggressive behavior, and the modifiers that mediate these effects, will be essential to understand the molecular mechanisms underlying intergenerational and transgenerational epigenetic inheritance. This together with emerging technologies will complement human genome-wide epigenetic studies to improve our understanding of epigenetic programming in the germline and during preimplantation development and will have wide-ranging implications for human health. Whatever the experimental model chosen and the types of prenatal maternal stress studies, (whether trauma, psychological, or physical), there is need for restrain before claiming a heritable transgenerational response. There is need also to demonstrate the occurrence of epigenetic modification and to associate these modifications to aggressive behavior in the offspring. Demonstrating the presence of aggressive behavior and the altered epigenetic marks in the F2 and F3 generation as well-understanding the similarities or differences in the transfer mechanisms from one generation to the other will be necessary. As more studies investigate and associate the etiology and biological correlates of aggressive behavior in F2 and F3 generations with intergenerational stress, it will be necessary to determine under what conditions these persist or disappear. While observations have been made in studies of offspring prenatally exposed to natural disasters, genocides, and wars, where offspring of affected parents showed higher rates of depression and psychopathological disorder symptoms (Huizink et al., 2007), very few studies have directly correlated intergenerational stress and aggressive behavior in offspring. One study at least suggests that stress is transferred to a second generation and sons and daughters of holocaust survivors were less likely to externalize aggression than were those in a control group (individuals who were socialized by parents who did not suffer the Holocaust directly) (Nadler et al., 1985).

Given growing evidence establishing the intergenerational transmission of stress from mother to offspring despite divergent views on the mechanisms, it will be vital to find out whether there are factors that attenuate or stop such transmissions and, further, to identify these attenuating factors. There is scanty information relating to the modulation of the HPA axis during pregnancy and its benefits, (Urizar and Muñoz, 2011), however suggests that stress reduction activities reduce maternal perceived stress as well as morning cortisol levels. The early postpartum period also may be critical and provide a window to allow for the modification of *in utero* programmed changes. An animal studies have shown that postnatal care also influences offspring HPA axis physiology. This is associated with augmented sensitivity of the axis to negative feedback, reduced CRF and diminished stress response, which accordingly reduces anxiety and related behavior in offspring (Gunnar and Quevedo, 2008; Roberts and Lopez-Duran, 2019). This presents opportunity for instituting early detection of, and prevention of potential psychopathological disorders.

This review preferentially explored intergenerational and transgenerational transmission of stress from the perspective of prenatal maternal exposure to trauma on the potential of offspring aggressive, before and during pregnancy. It does not consider the contributions of paternal stress across germline. Also, the lack of consensus on the definitions of epigenetics, intergenerational and transgenerational transmission as well as methodological challenges, the comprehension and presentation of intergenerational or transgenerational transmission of stress makes it complicating to provide a comprehensive summary of advances in the field. Despite the limitations, the studies examined in this review, all point to the susceptibility of the fetal brain to prenatal maternal stress and an adverse maternal environment during pregnancy through mechanisms that are associated and potentially even mediated by epigenetic regulations. These changes may provide a molecular basis for the aggressive traits observed in offspring, suggesting the transcriptional potential prenatal stress has and how long lastingly responses to these can have on aggression and behavior in general. The extent to which prenatal maternal stress contribute to the development of aggressive behavior in later generations of offspring is still far from being understood.

## Author contributions

NM conceived and wrote the first draft. SH and LQ reviewed and made significant input. All authors contributed to the article and approved the submitted version.
